# Height status matters for risk of mortality in critically ill children

**DOI:** 10.1186/s40560-024-00757-9

**Published:** 2024-10-29

**Authors:** Nobuyuki Nosaka, Tatsuhiko Anzai, Kenji Wakabayashi

**Affiliations:** 1 Department of Intensive Care Medicine, Graduate School of Medical and Dental Sciences, Institute of Science Tokyo, 1-5-45 Yushima, Bunkyo-Ku, Tokyo, 113-8510 Japan; 2 Department of Biostatistics, M&D Data Science Center, Institute of Integrated Research, Institute of Science Tokyo, Tokyo, Japan

**Keywords:** Mortality, Length of stay, Pediatric critical care, Stature

## Abstract

**Background:**

Anthropometric measurements are crucial in pediatric critical care, but the impact of height on ICU outcomes is underexplored despite a substantial number of short-for-age children in ICUs. Previous studies suggest that short stature increases the risk of poor clinical outcomes. This study examines the relationship between short stature and ICU outcomes.

**Methods:**

We conducted a retrospective cohort study using a Japanese nationwide database (the Japanese Intensive Care Patient Database; JIPAD), which included pediatric patients under 16 years admitted to ICUs from April 2015 to March 2020. Height standard deviation scores (SD scores) were calculated based on age and sex. Short-stature patients were defined as height SD score <  − 2. The primary outcome was all-cause ICU mortality, and the secondary outcome was the length of stay in ICU.

**Results:**

Out of 6,377 pediatric patients, 27.2% were classified as having short stature. The ICU mortality rate was significantly higher in the short-stature group compared to the normal-height group (3.6% vs. 1.4%, *p* < 0.01). Multivariable logistic regression showed that short stature was independently associated with increased ICU mortality (OR = 2.73, 95% CI 1.81–4.11). Additionally, the Fine–Gray subdistribution hazards model indicated that short stature was associated with a lower chance of ICU discharge for each additional day (HR 0.85, 95% CI 0.81–0.90, *p* < 0.01).

**Conclusions:**

Short stature is a significant risk factor for increased ICU mortality and prolonged ICU stay in critically ill children. Height should be considered in risk assessments and management strategies in pediatric intensive care to improve outcomes.

## Introduction

Anthropometry on admission to the intensive care unit (ICU) is commonly used in evaluating children’s nutritional status and identifying those at risk of suboptimal clinical outcomes. Low height status, or short stature, is an important indicator reflecting chronic malnutrition in children [1, 2]. Notably, previous studies reported a significant prevalence of short stature ranging from 16 to 50% in children requiring intensive care [3–9]. Moreover, impaired height growth is a classical but important risk factor for mortality [10]. Additionally, previous studies reported that pediatric short stature was associated with various clinical outcomes including treatment failure of pneumonia [11], prolonged duration of mechanical ventilation [4, 5], and extended length of ICU stay [6] or hospital stay [7]. These findings suggest that height status impacts the prognosis of ICU children and is a sensitive predictor of clinical outcomes, particularly where short stature is prevalent. However, these previous studies were conducted on non-Japanese pediatric populations, who are generally taller and heavier than Japanese children [12]. Moreover, knowledge regarding the relationship between short stature and clinical outcomes in the overall pediatric ICU population remains limited. These observations prompted us to investigate the association between short stature and pediatric ICU outcomes, including mortality and the length of ICU stay, using the Japanese nationwide database.

## Methods

### Study design and cohort

We performed a retrospective cohort study using the same data as our previously published study [9] derived from the Japanese Intensive Care Patient Database (JIPAD). JIPAD is the largest national ICU registry in Japan [13]. We obtained the 5-year data of pediatric patients aged less than 16 years who were admitted to ICU from April 2015 to March 2020. The data obtained from JIPAD included patient demographics (sex and age), anthropometric measurements (bodyweight and height), clinical variables (admission category, elective/emergency admission status, reason for ICU admission, readmission during the same hospitalization, and Pediatric Index of Mortality 2 [PIM2] scores) [14], and outcomes (ICU mortality and length of stay [LOS] in ICU). For the entire dataset, we obtained age- and sex-adjusted SD scores for height (HT-SDS) and body mass index (BMI-SDS), which were calculated using the Excel-based Clinical Tool for Growth Evaluation of Children provided by the Japanese Society for Pediatric Endocrinology (JSPE: A general version can be downloaded at http://jspe.umin.jp/medical/chart_dl.html, Accessed on April 2021. A special form for big data analysis was provided courtesy of Dr. Yoshiya Ito on behalf of JSPE). Each SD score demanded age-in-month to be calculated. However, the JIPAD provides age-in-year for subjects aged more than 3 years. Thus, for subjects aged 3 years or older, we computed SD scores employing a surrogate age-in-month of “12 × (age) + 6” as previously described [9]. Subjects were excluded if they had missing or improbable anthropometric data, or anthropometric outliers defined as BMI-SDS <  − 5 or > 5 [15], or if their admission category was recorded as “operative procedures in the ICU”, or if they were readmission cases within the same hospitalization.

### Definition of anthropometric categories

Subjects were firstly classified into two height categories according to height status (short stature and normal height). Short stature was defined as height SD score (HT-SDS) <  − 2 [16, 17]. Subjects were also classified according to BMI categories. There are 4 BMI categories based on BMI-SDS: BMI-SDS <  − 2 to define underweight, − 2 ≦ BMI-SDS < 1 to define normal weight, 1 ≦ BMI-SDS < 2 to define overweight, and BMI-SDS ≧ 2 to define obesity [18, 19].

### Outcomes

The primary outcome of this study was all-cause ICU mortality. The secondary outcome was the LOS in ICU.

### Statistical analysis

All statistical analyses were performed using EZR (Saitama Medical Center, Jichi Medical University, Saitama, Japan), which is a graphical user interface for R (The R Foundation for Statistical Computing, Vienna, Austria) [20]. The description of the study population such as demographic, anthropometric, clinical, and outcome variables is presented as a frequency or the median with the interquartile range (IQR), as appropriate, with bivariate associations with height status category tested by using chi-square analysis or Wilcoxon rank test, respectively. Continuous variables that were normally distributed such as HT-SDS and BMI-SDS are documented as means (± standard deviations). Expected numbers of mortality were also calculated using PIM2 [21].

We performed multivariable logistic regression analysis to determine the association of height categories with all-cause ICU mortality controlling potential confounders. The potential confounders considered for inclusion in the model were age, sex, admission categories (status post-elective surgery, status post-emergency surgery, non-surgical), emergency admission, reasons for ICU admission (cardiovascular disease, respiratory disease, gastrointestinal disease, neurological disease, sepsis, cardiopulmonary arrest, and others), BMI status, and PIM2 scores. We next performed Gray’s test to describe LOS in ICU between the normal-height group and the short-stature group. Additionally, we performed the Fine–Gray subdistribution hazards model for multi-factor analysis using the same covariates shown above to determine the association of short stature with LOS in ICU while accounting for the competing events of death. These analyses were restricted to the cases with complete datasets.

### Ethics

This study was reviewed and approved by the Tokyo Medical and Dental University Review Board (M2020-245) and the JIPAD steering committee. The need for informed consent was waived considering the retrospective design and complete anonymization. The anonymized data were provided for analysis by the JIPAD. The study was performed following the relevant guidelines and regulations.

## Results

### Patient characteristics

**Fig. 1:**
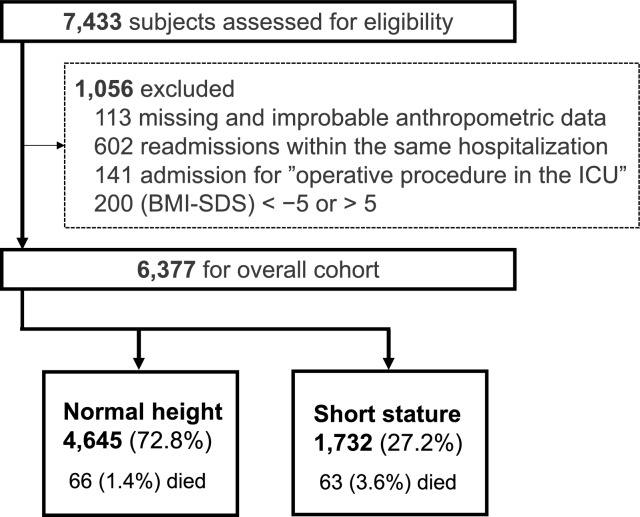
Cohort creation flowchart

There were 7,433 consecutive patient records identified in the JIPAD between the inclusive study dates, and 6,377 had complete data for anthropometric data and met the study inclusion criteria (Fig. 1). Short-stature subjects account for as much as 27.2% of the cohort.

The demographic characteristics are described in Table 1. Although most between-height category differences were statistically significant due to the large sample size, some of these differences may not be clinically relevant. However, the median PIM2 score was higher in the overall short-stature group than the normal-height group (median: 1.2 [0.4–2.6] vs. 0.8 [0.2–1.9], *p* < 0.01). The estimated mortality calculated by PIM2 was 69.9 (4.0%) in the short-stature group and 144.4 (3.1%) in the normal height group (*p* = 0.08).

**Table 1:** Demographic and clinical characteristics in children admitted to intensive care unit

	Overall	Short stature	Normal height	*p* value
Subjects, No. (%)	6,377	1,732 (27.2)	4,645 (72.8)	
Age (year), median [IQR]	2 [0, 8]	2 [0, 6]	3 [0, 8]	< 0.01^†††^
Female sex, No. (%)	2,913 (45.7)	829 (47.9)	2,084 (44.9)	0.04^†^
Reason for ICU admission, No. (%)				< 0.01^††^
Cardiovascular	1,724 (27.0)	511 (29.5)	1,213 (26.1)	
Respiratory	1,476 (23.1)	468 (27.0)	1,008 (21.7)	
Neurological	1,391 (21.8)	241 (13.9)	1,150 (24.8)	
Gastrointestinal	735 (11.5)	251 (14.5)	484 (10.4)	
Cardiopulmonary arrest	84 (1.3)	25 (1.4)	59 (1.3)	
Sepsis	39 (0.6)	17 (1.0)	22 (0.5)	
Others	928 (14.6)	219 (12.6)	709 (15.3)	
Emergency admission, No. (%)	2,279 (35.7)	648 (37.4)	1,631 (35.1)	0.09^†^
Admission category, No. (%)				< 0.01^††^
Post-elective surgery	4,061 (63.7)	1,079 (62.3)	2,982 (64.2)	
Post-emergency surgery	494 (7.7)	93 (5.4)	401 (8.6)	
Non-surgical	1,822 (28.6)	560 (32.3)	1,262 (27.2)	
BMI SD score, mean (SD)	−0.44 (1.52)	−0.62 (1.85)	−0.37 (1.37)	< 0.01
BMI status, No. (%)				< 0.01^††^
Underweight	931 (14.6)	396 (22.9)	535 (11.5)	
Normal weight	4,433 (69.5)	1,026 (59.2)	3,407 (73.3)	
Overweight	722 (11.3)	178 (10.3)	544 (11.7)	
Obese	291 (4.6)	132 (7.6)	159 (3.4)	
Height SD score, mean (SD)	−1.15 (2.03)	−3.69 (1.66)	−0.20 (1.15)	< 0.01
PIM2, median [IQR]	0.9 [0.2, 2.1]	1.2 [0.4, 2.6]	0.8 [0.2, 1.9]	< 0.01^†††^
Estimated mortality by PIM2 (%)	214.3 (3.4)	69.9 (4.0)	144.4 (3.1)	0.08^†^
Length of ICU stay (day), median [IQR]	3.0 [2.0, 6.0]	3.0 [2.0, 7.0]	3.0 [2.0, 6.0]	< 0.01^†††^
ICU mortality (%)	129 (2.0)	63 (3.6)	66 (1.4)	< 0.01^†^

*BMI* body mass index, *ICU* intensive care unit, *IQR* interquartile range, *PIM2* Pediatric Index of Mortality 2, *SD* standard deviation

^†^chi-square test

^††^chi-square test for equal frequencies of all categories between groups

^†††^Wilcoxon rank test

### Primary outcome: all-cause ICU mortality

A total of 129 (2.0%) subjects in the overall cohort died in the ICU. All-cause ICU mortality was higher in the short-stature group compared with the normal-height group (3.6% vs. 1.4%, respectively, *p* < 0.01; Table 1).

To determine whether height status independently contributes to ICU mortality and to exclude potential confounders, we conducted a multivariable logistic regression analysis. In cases where anthropometric data were fully available, all adjustment variables were complete; however, mortality data were missing for three cases. Consequently, the subsequent analyses were performed using complete datasets comprising 6,374 subjects. The multivariable analysis revealed a statistically significant increase in ICU mortality for the short-stature group compared to the normal height reference group (OR = 2.73, 95%CI 1.81–4.11; Table 2). As a side note, we considered the possibility of a statistical interaction between BMI categories and height categories, but it was computed to be not significant.

**Table 2:** Variables associated with mortality in children admitted to intensive care unit

	Survived	Died	Univariate logistic regression analysis	Multivariable logistic regression analysis	
			OR (95% CI)	OR (95% CI)	*p* value
Subjects, No. (%)	6,245 (98.0)	129 (2.0)	NA	NA	NA
Height SD score, mean (SD)	−1.13 (2.01)	−2.08 (2.80)	NA	NA	NA
Height category, No. (%)					
Short stature	1,668 (26.7)	63 (48.8)	2.62 (1.85–3.72)	2.73 (1.81–4.11)	< 0.01
Normal height	4,577 (73.3)	66 (51.2)	Reference	Reference	
Age (year), median [IQR]	2.0 [0.0, 8.0]	1.0 [0.0, 7.0]	0.97 (0.93–1.01)	1.01 (0.96–1.05)	0.80
Female sex, No. (%)	2,846 (45.6)	66 (51.2)	1.25 (0.88–1.77)	1.08 (0.73–1.59)	0.71
Reason for ICU admission, No. (%)					
Cardiovascular	1,680 (26.9)	42 (32.6)	2.87 (1.34–6.15)	5.15 (2.22–11.90)	< 0.01
Respiratory	1,451 (23.2)	25 (19.4)	1.98 (0.89–4.41)	1.40 (0.59–3.33)	0.45
Neurological	1,374 (22.0)	17 (13.2)	1.42 (0.61–3.31)	1.20 (0.49–2.96)	0.70
Gastrointestinal	723 (11.6)	11 (8.5)	1.75 (0.70–4.37)	2.23 (0.83–6.03)	0.11
Cardiopulmonary arrest	65 (1.0)	19 (14.7)	33.60 (14.20–79.70)	1.07 (0.36–3.17)	0.91
Sepsis	32 (0.5)	7 (5.4)	25.20 (8.60–73.60)	4.21 (1.11–15.90)	0.03
Others	920 (14.7)	8 (6.2)	Reference	Reference	
Emergency admission, No. (%)	2,176 (34.8)	101 (78.3)	6.75 (4.42–10.30)	1.14 (0.42–3.07)	0.80
Admission category, No. (%)					
Post-elective surgery	4,036 (64.6)	24 (18.6)	Reference	Reference	
Post-emergency surgery	473 (7.6)	19 (14.7)	6.76 (3.67–12.40)	5.74 (1.81–18.30)	< 0.01
Non-surgical	1,736 (27.8)	86 (66.7)	8.33 (5.28–13.10)	5.72 (2.09–15.60)	< 0.01
BMI SD score, mean (SD)	−0.43 (1.51)	−0.56 (1.95)	NA	NA	
BMI status, No. (%)					
Underweight	903 (14.5)	27 (20.9)	1.69 (1.08–2.64)	1.11 (0.67–1.84)	0.70
Normal weight	4,354 (69.7)	77 (59.7)	Reference	Reference	
Overweight	709 (11.4)	13 (10.1)	1.04 (0.57–1.88)	0.95 (0.49–1.84)	0.88
Obese	279 (4.5)	12 (9.3)	2.43 (1.31–4.52)	1.36 (0.64–2.86)	0.42
PIM2, median [IQR]	0.90 [0.20, 2.00]	14.20 [4.70, 54.90]	1.06 [1.05–1.06]	1.05 [1.04–1.06]	< 0.01

*BMI* body mass index, *CI* confidence interval, *ICU* intensive care unit, *IQR* interquartile range, *SD* standard deviation, *OR* odds ratio, *PIM2* Pediatric Index of Mortality 2

### Secondary outcome: LOS in ICU

**Fig. 2:**
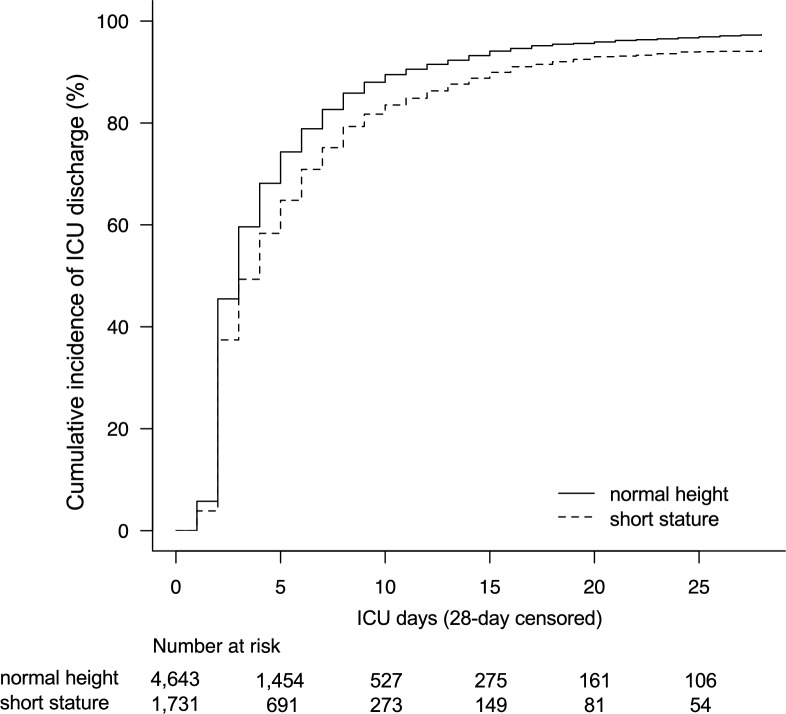
The cumulative incidence for length of stay in ICU (up to 28 days) in each height category

The median ICU LOS was 3 days (IQR; 2–6 days). The distribution of ICU LOS between the short-stature group and the normal-height group was statistically different (3.0 [IQR: 2.0–7.0] days vs. 3.0 [2.0–6.0] days, respectively, *p* < 0.01). The cumulative incidence for ICU LOS (up to 28 days) in each height category is depicted in Fig. 2 (*p* < 0.01). The Fine–Gray subdistribution hazard model demonstrated the significant association of height status with ICU LOS in this cohort (Table 3). For each additional day in the ICU, short-stature subjects had a 15% (hazard ratio [HR], 0.85; 95% CI 0.81–0.90; *p* < 0.01) lower chance of being discharged than normal-height subjects, after controlling for age, sex, admission categories, emergency admission, reasons for ICU admission, BMI categories, and PIM2 scores.

**Table 3:** The Fine-Gray subdistribution hazard model of variables associated with length of stay in intensive care unit among pediatric patients, *n* = 6,374

	Hazard Ratio (95% CI)	*p* value
Height category		
Short stature	0.85 (0.81–0.90)	< 0.01
Normal height	Reference	
Age (year)	1.02 (1.01–1.03)	< 0.01
Female sex	0.94 (0.90–0.99)	0.01
Reason for ICU admission		
Cardiovascular	0.54 (0.50–0.58)	< 0.01
Respiratory	0.92 (0.85–1.00)	0.04
Neurological	0.99 (0.92–1.07)	0.84
Gastrointestinal	0.96 (0.88–1.06)	0.43
Cardiopulmonary arrest	1.07 (0.85–1.36)	0.56
Sepsis	0.59 (0.42–0.82)	< 0.01
Others	Reference	
Emergency admission	1.09 (0.96–1.23)	0.20
Admission category		
Post-elective surgery	Reference	
Post-emergency surgery	0.53 (0.46–0.62)	< 0.01
Non-surgical	0.45 (0.40–0.51)	< 0.01
BMI status		
Underweight	0.88 (0.83–0.94)	< 0.01
Normal weight	Reference	
Overweight	1.04 (0.96–1.12)	0.37
Obese	1.02 (0.92–1.13)	0.77
PIM2	0.97 (0.97–0.98)	< 0.01

*BMI* body mass index, *CI* confidence interval, *ICU* intensive care unit, *PIM2* Pediatric Index of Mortality 2

For thoroughness, we repeated the two analyses including the three cases with missing mortality data by treating them as deceased as a sensitivity analysis (*n* = 6,377). There were no changes in the variables with statistical significance and in the direction of the estimates for each variable.

## Discussion

In this study, we examined the association between short stature and ICU outcomes including mortality and ICU LOS. We confirmed a high prevalence of short stature, as high as over one-quarter of children requiring intensive care. Our study found that being short, significantly increases the risk for ICU mortality of children requiring intensive care, even after adjustment for possible confounders within the scope of the available database. In addition, we showed that short-stature subjects had a lower chance of being discharged than normal-height subjects for each additional day in the ICU. Our study highlighted the importance of assessing height as an additional critical factor in evaluating the risk associated with pediatric patients in the ICU.

There are limited studies specifically addressing the prevalence and impact of short stature in ICU settings. A secondary analysis of pediatric oncology patients admitted to an ICU in China (*n* = 160) showed that 16.3% had short stature, correlating with a longer duration of mechanical ventilation [5]. A retrospective cohort study in a Brazilian pediatric ICU (*n* = 1,753) found that 23.6% had short stature [3]. A retrospective, single-center cohort study of children undergoing cardiac surgery in Singapore (*n* = 302) reported 26.8% had short stature, which was associated with longer hospital stays, mechanical ventilation, and increased inotrope use post-surgery [7]. A prospective, single-center cohort study of children undergoing cardiac surgery in the United Kingdom (*n* = 117) found that 28.5% of infants (≤ 12 months) and 20.6% of older children (1–16 years) had short stature, which was linked to a longer ICU LOS [6]. Another prospective cohort study in a Brazilian pediatric ICU (*n* = 72) found that 41.2% had short stature, also linked to a longer duration of mechanical ventilation [4]. In a tertiary ICU in Brazil, primarily attending to pediatric patients with chronic diseases (*n* = 90), 50% were reported to have short stature [8]. These findings collectively suggest that short stature prevalence varies with patient backgrounds, but remains significant and associated with unfavorable clinical outcomes in pediatric ICU admissions. Our study, utilizing a national ICU database with over 6,300 subjects, strongly reinforces the associations between short stature and poor clinical outcomes—especially mortality and ICU LOS—observed in previous smaller-scale single-center studies, which reaffirms the importance of height status assessment in pediatric intensive care.

Multiple studies have predominantly focused so far on the association between pediatric ICU outcomes and weight status as an indicator of nutritional status [19, 22–36]. Especially, BMI, calculated as weight (kg) divided by height squared (m^2^), has been shown as a valuable prognostic indicator for children in ICUs, aiding in the prediction of clinical outcomes such as mortality [27–33], the duration of mechanical ventilation [22, 27], and the length of ICU stay [27, 28, 34, 36]. However, the relationship between BMI status and clinical outcomes is inconsistent in other studies [3, 19, 36]. For instance, a systematic review by Toh et al. [19] found no significant association between BMI status and outcomes in critically ill children, including mortality, ICU LOS, hospital LOS, or duration of mechanical ventilation. Recently, we published a study that illustrated the distribution of height, weight and BMI among children admitted to ICUs in Japan using the same dataset as in this study [9]. The study demonstrated that the BMI-for-age distribution maintained a normal bell-shaped pattern while the distributions of height-for-age and weight-for age skewed towards the lower end. This indicates that the pediatric ICU population generally maintains a balanced physique but tends to be small for age. Lara-Pompa et al. [37] described similar anthropometric characteristics in children requiring hospital care, with poor agreement between BMI and height in nutritional status assessment. These discrepancies arise from the fact that BMI cannot distinguish between children with short stature and those of normal height. This means that among children with the same BMI, some could be short, while others might be of normal height [26]. Accordingly, in the population of children requiring intensive care with a high prevalence of short stature, height status may more accurately reflect the under-nutritional status. Indeed, in our analysis, height status in children was associated with ICU mortality, whereas BMI status was not. This supports the superiority of height status, compared to BMI, in identifying patients at risk of suboptimal clinical outcomes.

On the other hand, the etiology of short stature is not limited to undernutrition. It is highly multifaceted, ranging from physiological normal variants such as familial short stature to pathological causes like genetic/chromosomal, neonatal, skeletal, or hormonal diseases [16, 17, 38]. The effect of medications, such as steroids, may also be an important factor [38]. These various factors are intricately intertwined, and it cannot be denied that they may deeply influence the relationship between short stature and ICU outcomes. In particular, in the pediatric population requiring intensive care, the impact of malnutrition caused by metabolic stress response in conjunction with preexisting chronic diseases must be considered [2]. Children with complex chronic conditions (CCC) are those who suffer from one or more significant health issues that are expected to last at least 12 months, potentially resulting in functional limitations, dependency on medical technology, and frequent healthcare needs [39]. CCCs encompass a wide range of diagnoses, including chromosomal anomalies, complex congenital heart diseases, neurologic diseases, genetic disorders, and chronic respiratory diseases. The number of children with CCC is increasing, representing a growing proportion of patients in the ICU [40, 41]. Indeed, Edwards et al. [42] demonstrated that as many as 53% of PICU admissions involved CCC, and these children were at greater risk for ICU mortality and prolonged ICU LOS in a multicenter study in the United States involving 52,791 critically ill children. Furthermore, children with CCC have a higher prevalence of short stature. Rupp Hanzen Andrades et al. [3] reported that 32.2% of ICU children with CCC had short stature, which is higher than the 15.0% observed in those without CCC. Accordingly, the poor ICU outcomes associated with short stature may be closely related to CCC. At the same time, these facts underscore the importance of acknowledging the numerous unmeasured and potentially significant confounding factors that may explain the observed relationship between short stature and poor ICU outcomes, which were not accounted for in our study.

The PIM2 has been shown to have excellent discriminatory power and good calibration in Japanese children requiring intensive care, although it tended to overestimate the number of deaths [43, 44]. From this perspective, it seems reasonable that the estimated mortality calculated by PIM2 was higher than the observed mortality in the normal-height group. Conversely, in the short-stature group, the number of deaths was similar to the estimated mortality, which was expected to be an overestimate, leading to worse outcomes compared to the normal-height group. This relationship between PIM2 and observed mortality suggests a higher mortality risk in the short-stature group, underscoring the need for further investigation into the underlying causes.

The key strength of our study is the large size of cohort from the national ICU database. The dataset allowed us to conduct in-depth analyses, examining connections between short stature and short-term outcomes. This large dataset enabled us to eliminate potential confounding factors as much as possible and to minimize statistical bias. In addition, we calculated SDS of height and BMI based on Japanese local growth charts [45], which contributed to providing a more accurate anthropometric assessment of the studied populations. However, limitations still remain. First, there is a concern about unmeasured confounders regarding the etiology of short stature, such as familial socioeconomic status, chromosomal abnormality, birth history including birth weight/height and gestational age, and exposures from earlier stages of life. Additionally, in our analysis, we included the reason for ICU admission as one of the confounding factors, but underlying conditions, including CCCs, were not measured and could not be evaluated. Moreover, we were unable to evaluate how individual interventional factors affected the study outcomes. Furthermore, there may be variations among hospitals in the care provided to short-stature children. The second limitation is the accuracy of height measurement, as seen in previous studies [9, 33, 36]. In our study, height measurement methods were not standardized, although obtaining precise and standardized height measurements may pose challenges in critically ill children [46]. Third, our findings might not be generalizable to ICU patients outside Japan. It is recognized that there are noteworthy variations among countries in the definition of intensive care, patient demographics, admission and discharge practices, severity of illness, mortality rates, and LOS [47]. Specifically, it is worth noting that the proportion of obesity, which has been pointed out to influence ICU outcomes, is significantly lower in our study population compared to data from other countries [28–30, 33, 34].

## Conclusions

In conclusion, short stature is associated with pediatric ICU mortality and prolonged LOS in the ICU. Including height status in the risk assessment using anthropometry for ICU children is crucial for a more comprehensive evaluation.

## Data Availability

The data that support the findings of this study are available from JIPAD, but restrictions apply to the availability of these data, which were used under permission for the current study, and are thus not publicly available. However, data are available from the authors upon reasonable request and with permission of the steering committee of JIPAD.

## Abbreviations

BMI: Body mass index
BMI-SDS: BMI standard deviation score
CCC: Complex chronic condition
CI: Confidence interval
HT-SDS: Height standard deviation score
HR: Hazard ratio
ICU: Intensive care unit
IQR: Interquartile range
JIPAD: The Japanese Intensive Care Patient Database
LOS: Length of stay
OR: Odds ratio
PIM2: Pediatric Index of Mortality 2
PRISM: The pediatric risk of mortality
SD(S): Standard deviation (scores)
